# A Novel Hospital-to-Home System for Children With Medical Complexities: Usability Testing Study

**DOI:** 10.2196/34572

**Published:** 2022-08-12

**Authors:** Marissa Bird, Nancy Carter, Audrey Lim, Nadia Kazmie, Cindy Fajardo, Shannon Reaume, Michael H McGillion

**Affiliations:** 1 School of Nursing McMaster University Hamilton, ON Canada; 2 Department of Pediatrics McMaster University Hamilton, ON Canada; 3 Ontario Health (OTN) Toronto, ON Canada; 4 School of Public Health and Health Systems University of Waterloo Waterloo, ON Canada; 5 Population Health Research Institute Hamilton, ON Canada

**Keywords:** usability testing, digital health, children with medical complexities, children, chronic disease, pediatrics, health care, parenting, virtual health, care provider, youth, family needs, home care, usability

## Abstract

**Background:**

Children with medical complexity (CMC) are a group of young people who have severe complex chronic conditions, substantial family-identified service needs, functional limitations, and high health care resource use. Technology-enabled hospital-to-home interventions designed to deliver comprehensive care in the home setting are needed to ease CMC family stress, provide proactive and comprehensive care to this fragile population, and avoid hospital admissions, where possible.

**Objective:**

In this usability testing study, we aimed to assess areas of strength and opportunity within the DigiComp Kids system, a hospital-to-home intervention for CMC and their families and care providers.

**Methods:**

Hospital-based clinicians, family members of medically complex children, and home-based clinicians participated in DigiComp Kids usability testing. Participants were recorded and tasked to think aloud while completing usability testing tasks. Participants were scored on the metrics of effectiveness, efficiency, and satisfaction, and the total usability score was calculated using the Single Usability Metric. Participants also provided insights into user experiences during the postusability testing interviews.

**Results:**

A total of 15 participants (5 hospital-based clinicians, 6 family members, and 4 home-based clinicians) participated in DigiComp Kids usability testing. The participants were able to complete all assigned tasks independently. Error-free rates for tasks ranged from 58% to 100%; the average satisfaction rating across groups was ≥80%, as measured by the Single Ease Question. Task times of participants were variable compared with the task times of an expert DigiComp Kids user. Single Usability Metric scores ranged from 80.5% to 89.5%. In qualitative interviews, participants stressed the need to find the right fit between user needs and the effort required to use the system. Interviews also revealed that the value of the DigiComp Kids system was in its ability to create a digital bridge between hospital and home, enabling participants to foster and maintain connections across boundaries.

**Conclusions:**

Usability testing revealed strong scores across the groups. Insights gained include the importance of tailoring the implementation of the system to match individual user needs, streamlining key system features, and consideration of the meaning attached to system use by participants to allow for insight into system adoption and sustainment.

## Introduction

### Background

Children with medical complexities (CMCs) have a significant disease burden, including neurological impairment and organ dysfunction [1]. Medical advances have led to many CMC living longer than would previously have been possible with the added support of technologies such as ventilators and feeding tubes [2]. International epidemiological data indicate that CMC commonly constitute <1% of all children in a given population [1,3,4]; however, Canadian data show that this fraction of the population accounts for up to one-third of pediatric health-related expenditures [1]. Within the current care model, CMC have, on average, 5 or more inpatient hospital stays per year, with a median of 38 days [5]. In addition to the expertise of their acute care hospital teams and specialists, families of technology-dependent CMC often require specialized home care nursing [6]; however, health professional support for this population is fragmented in terms of home and specialist care, with considerable variation in the provision of these services by region. Many regions lack adequate numbers of specialized pediatric home care nurses trained to care for CMC, and specialist care is often episodic, separated geographically from hospital and home care, and lacks integration in terms of communication and documentation with other care systems [6,7].

To reduce poor outcomes such as unmet health care needs as well as emergent and repeated hospitalizations of CMC, care models are needed that emphasize care coordination (organized care with a clear division of responsibility [5]), timely access to urgent care, and a focus on proactive, comprehensive care, as opposed to care that is reactive and episodic [8]. In other complex populations such as frail older people and adults coping with cancer, hospital-to-home models of care using technology-enabled digital health care systems have successfully been used to decrease unmet health needs and unplanned hospitalizations [9,10].

Although technology-enabled digital health care systems can theoretically help bridge the gap between hospital and home and improve outcomes in CMC, this area remains largely underexplored for this population. Despite the relatively low number of studies in this area, research has shown promise in easing the burden of care on CMC families and reducing poor outcomes such as reducing urgent and in-person health care delivery. For example, preliminary data from a virtual hospital-to-home intervention for CMC consisting of vital sign monitoring and virtual communication with a hospital-based clinical team showed a 42% reduction in emergency department visits per patient per month and a 26% reduction in inpatient admissions, with a 95% patient satisfaction rating [11]. Another virtual intervention program involving unrestricted access to a specialist health care team for parents of CMC via telephone, email, telemedicine, and in-person consultations demonstrated an increase in total health system encounters but a decrease in in-person home and clinic care [12]. These early studies demonstrate that virtual care models can indeed improve outcomes for CMC; however, the lack of scalable, standardized virtual care models, despite these promising results, suggests that a deeper exploration of factors influencing adoption, scalability, and spread is warranted.

### Usability Testing

Usability testing studies assist in understanding the interactions between people and technology to investigate the ease of use, learnability, and perceived benefits and challenges of novel systems according to diverse end user groups (those for whom a technology or product is ultimately designed) [13]. The granularity of these studies provides detailed usability information that informs larger concepts such as intervention adoption, scale, and spread. The metrics of effectiveness, efficiency, and satisfaction are widely accepted as important components in the composite concept of usability and should be incorporated into usability measurement and reporting [14]. In addition, qualitative user experience data provide valuable insight into user behaviors, perspectives, needs, and desired outcomes from technology systems, helping to inform the relevance and acceptance of the technology by end users [15]. In this study, we aimed to investigate the usability of a virtual hospital-to-home health system for CMC and their families, called DigiComp Kids (Cloud DX technology).

### DigiComp Kids Intervention

The DigiComp Kids intervention uses the Cloud DX Connected Health System and consists of a hospital clinician portal and a home-based kit designed to communicate with one another. The hospital clinician portal is intended to enable hospital-based clinical teams to review biometric data and health information submitted by families and home-based clinicians as well as to send health information to home-based kits to facilitate home-based care management decision-making. Hospital-based clinicians can review submitted patient vital sign measurements, photos, and survey responses; configure individual vital sign parameters and alerts for each patient; send and receive secure messages with family members and home-based clinicians; send health-related documents to families and home-based clinicians such as care plans or medication schedules; schedule and initiate video calls with families and home-based clinicians; and document patient care information directly within the hospital clinician portal. The home-based kit is intended for use by CMC family members and their home-based clinicians to transmit biometric data and health information to hospital-based clinical teams as well as to receive health information sent by hospital-based clinical teams. Components of the home-based kit include a Samsung tablet, a Bluetooth-enabled pulse oximeter with heart rate monitoring capabilities, and a dual tympanic-temporal infrared thermometer. Kit components facilitate remote monitoring of biophysical parameters such as body temperature, heart rate, oxygen saturation, and respiratory rate (manual measurement); submission of responses to health-related monitoring questions; direct upload of photos to the cloud-based patient chart; real-time connection with hospital-based providers via video link and secure text messaging; and virtual appointment scheduling.

The DigiComp Kids system was designed with hospital-based clinicians, CMC families, and home-based clinicians to allow for comprehensive team-based care for CMC in the home setting. Details of the design methodology are available in detail in a previously published manuscript [16]. The aim of the DigiComp Kids system is to connect hospital-based clinical teams with CMC families and their home clinicians to proactively monitor CMC health needs and respond to them in a timely manner. By better connecting families with home- and hospital-based clinicians, the DigiComp Kids system has been designed to facilitate safe care at home for CMC.

### Objectives

The objective of this usability testing study was to assess areas of strength and opportunity within the DigiComp Kids system according to hospital-based clinicians, medically complex children and their family members, and home-based clinicians. During this early formative stage, the results from this usability testing study will assist in making further improvements to the DigiComp Kids system during the preclinical implementation phase.

## Methods

### Ethics Approval

The Hamilton Integrated Research Ethics Board (HiREB Project #8324) approved this study. All participants provided informed consent before engaging in usability testing.

### Setting, Recruitment, and Participant Groups

Usability testing took place entirely virtually because of the need for physical distancing and research regulations in place during the COVID-19 pandemic. In usability testing studies, the engagement of 4 or more participants per group is typically sufficient to detect >80% usability problems [17]; thus, we recruited 6 family members, 4 home-based clinicians, and 5 hospital-based clinicians, for a total of 15 usability testing participants. All participants resided in Southern Ontario, and usability testing was conducted between November and December 2020.

Hospital-based and home-based clinicians were recruited via the networks of various members of the study team (MB, NC, AL, MHM, and SR). Emails were sent to distribution lists and individual contacts known to be working with CMC in either hospital or home settings. The included clinicians spoke and read English, had at least 3 months of experience caring for CMC in hospital or home settings, and provided informed consent to participate. Hospital-based clinicians included a system navigator, complex care nurse practitioner, and 3 registered nurses working with CMC populations. Home-based clinicians included registered nurses and a registered practical nurse working directly with CMC in home settings as well as a clinical nurse specialist whose role is to support the provision of home care by offering remote clinical support.

Family participants were recruited by a clinical member of the study team (AL) and a CMC family partner on the study team (SR). The included family participants lived in the Southern Ontario area, had a child that met the definition of medical complexity [18], spoke and read English, and provided informed consent for both themselves and their child to participate. In this study, all recruited family members were mothers of medically complex children.

### Procedures

Our usability testing procedure incorporated both quantitative and qualitative measures via standardized usability testing and individual participant interviews to capture user effectiveness, efficiency, satisfaction, and user experience.

#### Training

##### Overview

All participants received DigiComp Kids intervention training using the Connected Health System through a dedicated virtual session. Before training, participants received either an at-home Connected Health System kit (family members and home-based clinicians) or access to the hospital Connected Health System clinician portal (hospital-based clinicians) as appropriate. All participants also received an electronic standardized training manual developed by the lead author of this study (MB) to guide the training session. The purpose of the training sessions was to orient participants to the DigiComp Kids program using the Connected Health System and its features and to allow participants to navigate the system and ask questions. Participants were given a general background on the project and the way that the home- and hospital-based systems interact, before being specifically trained on the relevant components for their group, as detailed in subsequent sections.

##### Hospital-Based Clinicians

Hospital-based clinicians were trained in the use of the hospital clinician portal. Training was guided by a standardized training manual that clinicians could refer to as needed throughout the training and testing sessions. Training topics included patient vital sign alert management and configuration; communication with families and home-based clinicians via secure chat messages and video calls; patient home-based care scheduling, including changes to care plans, medications, and required data entry from families or home-based clinicians; and direct documentation within the cloud-based platform. All tasks were demonstrated by the trainer (MB) via remote screen sharing, and participants were subsequently given the opportunity to practice tasks to solidify their information retention and application.

##### Families and Home-Based Clinicians

CMC family members and home-based clinicians were trained in the use of the at-home DigiComp Kids kit. Practice kits were delivered to family and home-based clinician participants before training. Similar to the hospital-based clinician training, home-based training was guided by a standardized training manual provided to participants for their use throughout the training and testing sessions. Topics for participant training included tablet log-in and setup; sending and receiving secure chat messages; viewing and undertaking scheduled vital signs assessments, photos, and surveys; and locating shared documentation such as care plans and medication orders. Participants were guided through tasks by the trainers (MB and NK) over video and were encouraged to follow along and participate with their kits before the testing session took place.

#### Testing

Testing for all participants was scheduled either immediately following or as soon as possible after the training sessions within a few days. All testing sessions were audio-visual recorded using videoconferencing software to facilitate the review and scoring of usability testing sessions at a later time.

##### Hospital-Based Clinicians

Following the training session, hospital-based clinicians participated in an individual virtual testing session facilitated by a moderator (MB). Testing sessions began by reminding the participants that usability testing was intended to test the DigiComp Kids system and approach usability training and not their performance or abilities as clinicians. Participants were given an opportunity to ask questions and were reminded that there would be a scheduled break during testing, but that they could request additional breaks at any time.

Next, participants were asked to think aloud during usability testing. Thinking aloud involves participants concurrently performing a task while verbalizing what comes to mind during the performance of that task [19]. The purpose of thinking aloud is for the moderator to gather relevant data on specific issues of usability, such as system navigation issues, areas of frustration or obscurity, or confusion around the workflow when using the system.

Hospital-based clinicians were asked to complete a series of usability testing tasks guided by a standardized testing protocol. Tasks within the protocol represented the core competencies for the hospital clinician portal, including locating patient information; responding to changes in patient vital signs; and communication, documentation, and scheduling of patient tasks such as surveys and video calls. Usability testing tasks for hospital-based clinicians are listed briefly in Textbox 1 and in further detail in Multimedia Appendix 1.

**User testing tasks for hospital-based clinicians.**

**Tasks**

- Task 1: Verbalize vital signs readings and any generated alerts for last assessment
- Task 2: Change Emma’s heart rate parameters from 0 (low) to 80 (high) bpm to 90 (low) to 130 (high) bpm
- Task 3: Request a video call with Emma’s family
- Task 4: Add a note to the oxygen saturation reading from this morning
- Task 5: Add assessment to chart with actions taken
- Task 6: Schedule a video call with Emma’s family in 4 hours
- Task 7: Change Emma’s risk stratification to “medium”
- Task 8: Change the “Wellness Survey” from being sent once weekly to being sent every day for 5 days
- Task 9: Send a chat message to Emma’s family

To add realism to the usability test, the participant tasks were conducted in the context of a simulated patient case. The fictional patient used for this case was a 2.5-year-old girl diagnosed with spinal muscular atrophy type 1, named Emma. Participants were introduced to Emma as their patient and provided clinical information on her condition, such as her main clinical issues (generalized low muscular tone and respiratory impairment) and the technical support used (portable oxygen and suction machine) to maintain her well-being at home. At prespecified time points, hospital-based clinicians were given new information about their patient case to indicate the progression of the patient scenario over time. Participants were asked to respond to information and updates on their patients given by the moderator throughout the testing process by following the clinical protocols that were taught during their training session. Further details of the patient are available in Multimedia Appendix 2.

##### Families and Home-Based Clinicians

CMC family members and home-based clinicians each participated in an individual usability testing protocol facilitated by a moderator (MB or NK). Think-aloud procedures were explained to the participants, as described earlier, and all participants were given the opportunity to ask questions before beginning their testing sessions. In contrast to the hospital-based clinician participants, usability testing sessions for family members and home-based clinicians did not take place within the context of a patient case but rather focused on undertaking day-to-day tasks related to caring for CMC using the DigiComp Kids home-based technology kit. During these sessions, family members and home-based clinicians were invited to imagine the use of a home-based technology kit with a medically complex child to which they provided care. For realism, family members were also given the option of applying peripheral vital sign devices (eg, pulse oximeter and thermometer) to their children, depending on their comfort level. The content of home-based clinician and family member usability testing sessions focused on device setup and log-in, peripheral vital sign device application and use, submission of clinical information such as responses to survey questions and photos to a simulated hospital-based clinical team, location of information sent by a simulated hospital-based team, and determination of required daily tasks using the scheduling function. Usability testing tasks for home-based clinicians and family members are listed briefly in Textbox 2 and in further detail in Multimedia Appendix 3.

**User testing tasks for family members and home-based clinicians.**

**Tasks**

- Task 1: Set up and turn on the tablet
- Task 2: Log-in to the tablet
- Task 3: View and interpret pending measurements and surveys using red asterisk
- Task 4: Complete or describe temperature measurement
- Task 5: Complete or describe oxygen saturation measurement
- Task 6: Complete and submit Wellness Survey
- Task 7: Find and read out the up-to-date list of child’s medications
- Task 8: Take and submit a photo using the tablet

#### Interviews

Immediately following the individual testing sessions, each participant was interviewed about their experience of participating in DigiComp Kids usability testing. The purpose of the qualitative data collection in this study was to improve our understanding of the usability of *DigiComp Kids* by triangulating quantitative usability metrics with user experience data from interviews [20,21]. A semistructured interview guide was developed using constructs from a holistic framework to improve the uptake and impact of eHealth technologies [21]. Specifically, the constructs used to guide the interview questions were those of “technology”—the hardware and software comprising the DigiComp Kids system; “people”—the participants themselves and other individuals specified by the participants; and context—the social, cultural, and physical environment in which the system is situated [21]. Across these categories, questions were designed to solicit areas of ease, frustration, and future improvement. Probing questions were used to encourage elaboration and clarification of participants’ responses where needed. Interview sessions were audio recorded using videoconferencing software. The interview guide can be found in Multimedia Appendix 4.

### Measures

**Figure 1:**
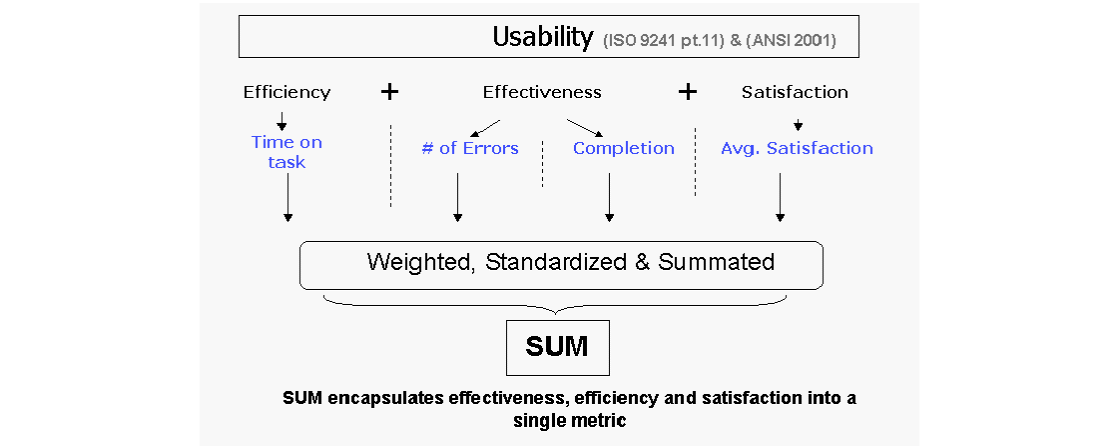
SUM model (reproduced from Sauro and Kinlund [22]). SUM: Single Usability Metric.

Usability is a composite measure comprising task completion, error rate, task time, and satisfaction score metrics [22]. Each of these measures was collected for each task that a user attempted during DigiComp Kids usability testing, with the goal of combining these measures into a Single Usability Metric (SUM) or SUM score [22] (Figure 1).

Task completion, error rates, task times, and satisfaction were calculated and standardized following the methods detailed by Sauro and Kinlund [22]. Task completion scores represented the ratio of successful task completion by participants to task attempts. Error rates were computed by dividing errors committed by the task error potential (number of participants multiplied by the number of subtasks per task) to account for multiple possible errors committed by the same participant on the same task. This value is subtracted from one to calculate the error-free rate, enabling it to be combined with other usability metrics into a summative score [22]. Task times and satisfaction scores were standardized by computing *Z* scores. To achieve this, a specification limit was set to represent an acceptable score. The specification limit for task time was the time it took an expert user to complete the task, multiplied by 1.5 [23], and for satisfaction, a value of 5.6 was used on the 7-point Single Ease Question satisfaction scale [24,25]. Detailed formulas for the usability measures can be found in Multimedia Appendix 5 [22-25].

### Data Management and Analyses

#### Quantitative Analysis

Microsoft Excel [26] v 16.3 was used for all quantitative statistical analyses. Descriptive statistics were used to summarize participants’ demographic data, child diagnostic information (family members), and professional work experience (clinicians).

In addition to reporting usability metrics by user group and task, Sauro and Kinlund [22] demonstrated that the constructs of efficiency, effectiveness, and satisfaction can be represented using a single SUM. Using SUM, usability as a construct is represented as a single score, making it intuitive to interpret without sacrificing the precision of using all 4 variables [22]. To construct the SUM, standardized metrics (task completion, error rates, task satisfaction, and task times) were averaged to create a single, summated score. This single score represents the overall usability of the DigiComp Kids system, equally weighted for the metrics of task completion, error rates, satisfaction, and task times.

#### Qualitative Analysis

Audiotaped interviews were transcribed verbatim, and the lead author (MB) proofread the transcripts to ensure accuracy. A qualitative descriptive approach was used to analyze the data. Initially, the 3 transcripts were read several times by 3 authors with qualitative training (MB, NK, and NC) before meeting to develop a coding scheme. The coding scheme was developed deductively using theoretical concepts from a holistic framework, including technology, people, and context [21]. This initial coding scheme was used to independently double-code 4 transcripts (MB and NK or MB and NC) using the Dedoose data management software and thematic analysis techniques [27,28]. Team members met to discuss preliminary findings and refine the coding structure. The remainder of the transcripts was coded by one author (MB or NK), and the authors met to discuss emerging themes.

Multiple measures were used to maintain rigor during the qualitative analysis. First, 1 author (NC) with qualitative expertise guided the qualitative data collection and analysis processes, approving methodological decisions before they were carried out. Second, all authors involved in the qualitative portion of the project (MB, NK, and NC) met weekly during the qualitative analysis process and were contacted via email between meetings to review the progress and discuss methodological issues. Process meetings were particularly helpful when major methodological milestones were encountered, for example, when defining and refining the code tree or when developing emerging themes. The process of peer review and triangulation of ideas helps establish confirmability in decisions [29]. Finally, a detailed audit trail was kept throughout the analysis process, documenting reflexive memos, meeting notes, coding and thematic decisions, and methodological processes.

## Results

### Demographics

#### Hospital-Based Clinicians

**Figure 2:**
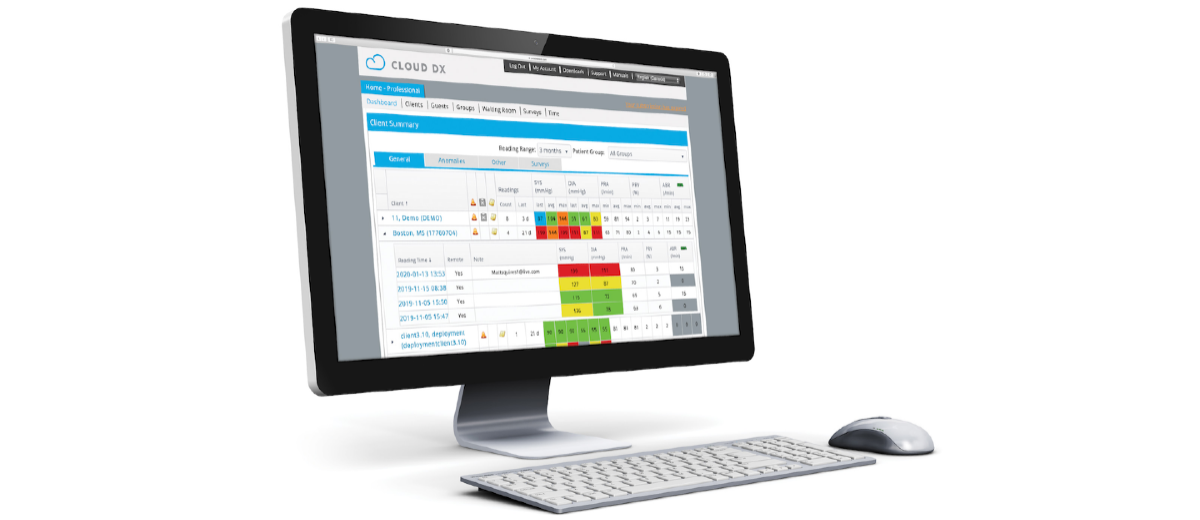
Clinician portal.

A total of 5 hospital-based clinicians participated in usability testing using the clinician portal (Figure 2). All participants were female, and the majority were employed full time and educated at a bachelor’s or graduate degree level. One participant was identified as a system navigator, and the rest were registered nurses or nurse practitioners. The average length of clinical practice among participants was 12 years, with 5 years practicing in CMC populations. The hospital-based clinician’s demographic variables are presented in Table 1.

**Table 1:** Hospital-based clinician characteristics (n=5).

Characteristics		Values
Gender (female), n (%)		5 (100)
**Ethnicity, n (%)**		
	Asian	1 (20)
	White	3 (60)
	Unspecified	1 (20)
**Role, n (%)**		
	Registered nurse	3 (60)
	Nurse practitioner	1 (20)
	System navigator	1 (20)
**Practice area, n (%)**		
	Pediatrics	2 (40)
	Complex care	3 (60)
Length of clinical practice (years), mean (SD)		12 (10.6)
Length of clinical practice with complex populations (years), mean (SD)		5 (2.3)

#### Family Members and Children

**Figure 3:**
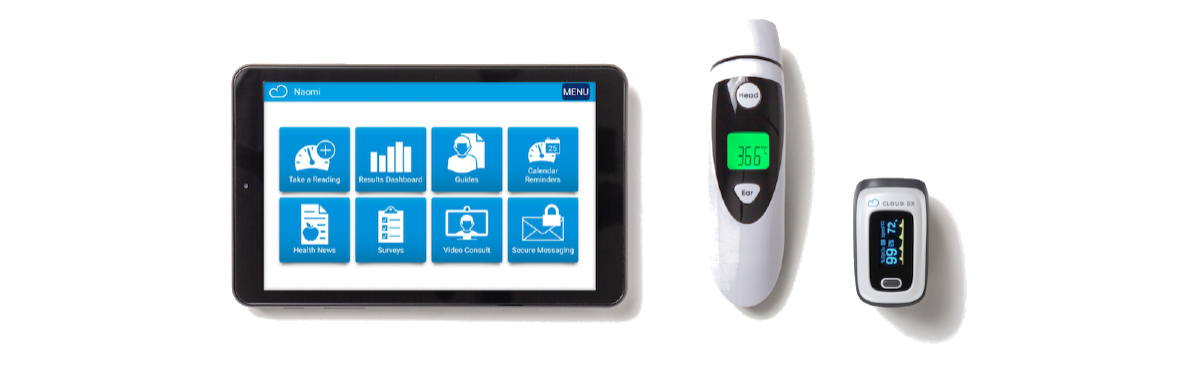
Components of the home-based kit.

A total of 6 family members participated in usability testing using the Connected Health Kit (Figure 3). Overall, 4 CMC participated in usability testing with their family members, and 2 family members simulated usability testing tasks because their children were unavailable during the testing time. All participating family members were female, and most were White and married. Most family members were educated at university level and working full time or on leave. The experience level of family members using tablet technology was evenly distributed from “somewhat experienced” to “expert.” The medically complex children of family members in this study were mostly male and White and aged between 2 and 7 years. CMC had 4 to 6 diagnosed chronic conditions and relied on a wide range of assistive technologies for support, as presented in Table 2.

**Table 2:** Family member and child characteristics.

Characteristics					Values, n (%)
**Family members (n=6)**					
	Gender (female)			6 (100)	
	**Ethnicity**				
		Asian	1 (17)		
		White	5 (83)		
	**Number of children**				
		1	1 (17)		
		2	2 (33)		
		3	1 (17)		
		4	2 (33)		
	**Highest level of formal education**				
		College diploma	1 (17)		
		Bachelor’s degree	3 (50)		
		Professional degree	1 (17)		
		Postgraduate certificate	1 (17)		
	**Employment status**				
		Unemployed	1 (17)		
		Part-time	1 (17)		
		Full-time	2 (33)		
		On leave	2 (33)		
	**Experience using tablet technology**				
		Somewhat experienced	2 (33)		
		Very experienced	2 (33)		
		Expert	2 (33)		
**Children (n=6)**					
	**Gender**				
		Female	2 (33)		
		Male	4 (66)		
	**Ethnicity**				
		Asian	1 (17)		
		White	5 (83)		
	**Age (years)**				
		2-4	3 (50)		
		5-7	3 (50)		
	**Number of chronic conditions**				
		4	2 (33)		
		5	2 (33)		
		≥6	2 (33)		
	**Assistive technology**				
		Enteral or parenteral feeding tube	5 (83)		
		Home oxygen	5 (83)		
		Mobility devices	3 (50)		
		Noninvasive ventilation	3 (50)		
		Invasive ventilation	2 (33)		
		Tracheostomy	2 (33)		
		Cerebrospinal fluid shunt	1 (17)		
		Long-term intravenous line or port	1 (17)		
		Communication devices	1 (17)		

#### Home-Based Clinicians

A total of 4 home-based clinicians participated in the usability testing. All home-based clinicians were White women who were employed in contract or part-time positions by community agencies or CMC families. The average length of clinical practice among participants was 14 years, with 12 years spent practicing CMC populations. The demographic characteristics of home-based clinicians are presented in Table 3.

**Table 3:** Home-based clinician characteristics.

Home-based clinicians (n=4)		Values
Gender (female), n (%)		4 (100)
Ethnicity (White), n (%)		4 (100)
**Role, n (%)**		
	Registered nurse	2 (50)
	Registered practical nurse	1 (25)
	Clinical nurse specialist	1 (25)
**Practice area, n (%)**		
	Complex care	3 (75)
	Home or community care	3 (75)
	Emergency department	1 (25)
	Neonatal intensive care	1 (25)
Length of clinical practice (years), mean (SD)		14 (16.0)
Length of clinical practice with complex populations (years), mean (SD)		12 (16.9)

### User Performance

The scores for task completion, error-free task rates, task satisfaction, and task times are presented by the end user group in Tables 4-6. SUM scores are presented for each task as well as the overall score per user group.

**Table 4:** Hospital-based clinician performance.

	Completion	Error-free rate	Satisfaction	Time	Single Usability Metric score
Task 1	1.00	0.80	0.9660	0.5675	0.8334
Task 2	1.00	0.85	0.9963	0.7549	0.9003
Task 3	1.00	1.00	0.9963	0.5199	0.8791
Task 4	1.00	0.97	0.9990	0.9878	0.9884
Task 5	1.00	0.93	0.8980	0.7580	0.8973
Task 6	1.00	0.92	0.8665	0.9798	0.9416
Task 7	1.00	1.00	0.9663	0.9999	0.9991
Task 8	1.00	0.70	0.3400	0.4350	0.6188
Task 9	1.00	1.00	0.9990	0.9994	0.9996
Score, mean (SD)	1.00 (0)	0.9078 (0.105)	0.8919 (0.212)	0.7780 (0.227)	0.8953 (0.119)

**Table 5:** Family participant performance.

	Completion	Error-free rate	Satisfaction	Time	Single Usability Metric score
Task 1	1.00	1.00	0.9987	0.6915	0.9226
Task 2	1.00	1.00	0.8577	0.3613	0.8048
Task 3	1.00	1.00	0.9987	0.9946	0.9983
Task 4	1.00	0.98	0.9222	0.8531	0.9392
Task 5	1.00	0.94	0.9987	0.9545	0.9744
Task 6	1.00	1.00	0.7734	0.6879	0.8653
Task 7	1.00	0.78	0.2327	0.3936	0.6010
Task 8	1.00	0.89	0.9806	0.4880	0.8394
Score, mean (SD)	1.00 (0)	0.9487 (0.079)	0.8453 (0.261)	0.6780 (0.246)	0.8681 (0.127)

**Table 6:** Home-based clinician performance.

	Completion	Error-free rate	Satisfaction	Time	Single Usability Metric score
Task 1	1.00	0.94	0.6879	0.2358	0.7153
Task 2	1.00	1.00	0.9893	0.9099	0.9748
Task 3	1.00	1.00	0.8159	0.2148	0.7577
Task 4	1.00	1.00	0.9990	0.8708	0.9675
Task 5	1.00	1.00	0.6664	0.7486	0.8538
Task 6	1.00	1.00	0.9990	0.5636	0.8909
Task 7	1.00	0.67	0.6844	0.3121	0.6658
Task 8	1.00	0.58	0.6554	0.2420	0.6202
Score, mean (SD)	1.00 (0)	0.8987 (0.172)	0.8058 (0.160)	0.5122 (0.298)	0.8057 (0.136)

DigiComp Kids usability testing revealed strong usability across end user groups with respect to task completion, errors, end user satisfaction, and task time. The average total SUM for hospital-based clinicians was 89.53%; family participants scored 86.81% across tasks, whereas home-based clinicians scored 80.57%. In terms of the individual score components, participants in all groups achieved task completion scores of 100%. Error rates varied (range 58%-100% error free), with participants achieving perfect scores across some tasks, whereas other tasks proved more complex and drew many errors. In general, participants committed more errors on tasks in which more steps were required to complete them (eg, task 8 for hospital-based clinicians). Participant satisfaction, as measured by the Single Ease Question, was generally high, with all groups, on average, reporting satisfaction scores of 80% or higher. A direct positive correlation was observed between the error-free rates and satisfaction scores. In general, simpler tasks (ie, those with fewer error opportunities) were more likely to be completed error free than more complicated tasks, and those tasks that were completed without errors by participants had higher satisfaction scores than those in which participants committed many errors. Task times varied across groups and were consistently the lowest scores of the 4 usability measures.

### Qualitative Findings

Thematic analysis of qualitative interview data generated 5 themes: fostering and maintaining team connections across boundaries; finding the right fit between user needs and required effort; improving system efficiencies and eliminating redundancies; making the system work in daily life; and reflecting on current and future technology needs.

#### Fostering and Maintaining Connections Across Boundaries

An important value highlighted in the co-design process for DigiComp Kids was that the system should aim to foster a sense of cohesion and connection between hospital-based clinicians, families, and home-based clinicians. During qualitative interviews, this aspect of the DigiComp Kids system was touched on by 12 of 15 participants, highlighting its importance. For example, 1 parent participant relayed:

Like, we were going to have an NG tube and we were going to come home with it and like having someone to walk us through, like doing those kinds of things that freak me out right now. I don’t want to do that... I don’t have the confidence of me to be listening to see if it went in. But if that [video call] was a possibility that would like totally ease my stress, like if she pulled out an NG tube that it would, um that somebody would be there to walk me through it.

Parent 002

For this participant, the possibility of remotely connecting with a clinician meant easing her stress while performing the procedure for her child at home. Similarly, hospital-based clinicians agreed that connecting with families at home would enable them to play a more supportive role for the child and the family:

I think that this also provides reassurance to families when they’re calling clinicians or emailing clinicians and they’re unsure of when they’re going to get a response. It might make families feel better. Even when it’s an initial discharge say from the NICU and a technology dependent kid and there’s a lot of anxiety on whether they’ll be able to reach clinicians or whether they live really far away geographically. They’ll have this one-on-one support depending on the hours.

Hospital 012

For families and care teams situated in different geographic areas, having access to a communication channel via a remote system can help break down boundaries and facilitate team cohesion and support.

#### Finding the Right Fit Between User Needs and Required Effort

Family members and hospital-based clinicians spoke of the need for the DigiComp Kids system to be implemented using flexible protocols that allow users to titrate their use of the system up or down as needed. Family members commented that CMC has labile medical conditions, resulting in a continuum of disease severity and subsequent health needs, depending on the manifestation of their conditions. The participants commented that the right fit between the user and the system would need to be struck to balance daily user requirements with self-identified user needs. One family member shared the importance of making the tool worthwhile to use:

Adding in another thing as another day-to-day task, that kind of seems like a bit much. If it was a point of time where we were trying to track something or a point where we were trying to wean him off the vent and we really wanted to zoom in on something, some numbers, I could see it being a daily thing... I’m trying to think of how it would be used as more tool rather than another chore, task to do with a complex kid.

Parent 001

Similarly, another family member spoke of the need for system use to add value to their lives as a motivation to adopt the system:

But having the unit open and on every day, unless it’s for a specific reason, it sounds really selfish but I feel like we would just open and use it if we needed something dealt with, not just so the team could find information of how normal our day is going. If that makes sense?

Parent 002

The fit or balance of required effort versus user needs was touched on by most participants as an important factor in whether they could envision adopting the DigiComp Kids system as part of their daily care routine.

#### Improving System Efficiencies and Eliminating Redundancies

In terms of improving system efficiency and eliminating redundancies, 5 participants spoke to the potential for the DigiComp Kids system to streamline and accelerate the timing of communication between clinicians. Using DigiComp Kids to enable multiple clinicians to view real-time patient data and communicate necessary changes in a timely fashion was seen as an important care improvement. For example, 1 home care clinician said:

Because [the hospital-based clinicians] are able to end up getting changes right away... rather than, for families what they would do is they would call the hospital, that they would page someone and then depending on how busy the person, [or] the team is, sometimes it takes a bit longer to answer that call or get back.

Home care 007

The DigiComp Kids system was seen as a strategy to accelerate necessary changes to care plans by allowing families, home-based clinicians, and hospital-based clinicians to view data in real time as patient changes are taking place. Other important factors for streamlining communication that arose in the interviews were ease of access to information and system interoperability, as detailed by the following participant:

I think this is so great. Honestly if everyone could just use this and have access to this, everyone’s life would be so much easier. Nurses, specialists, complex care teams, homecare, like I do not understand why we we’re are on so many different platforms for one patient.

Home care 009

Finally, participants also pointed out areas for improvement in the DigiComp Kids system that would further improve its usefulness with regard to streamlining team communication and work processes. For example:

When you have interdisciplinary groups, you’re like well I need this person to be able to address this issue and they might not be present at the time. I think that’s always the challenge in team communication, trying to get messages to people and you know in hospital, it’s like flagging charts and paging. So, if there was some way to flag people, that we need their attention, that would be really useful.

Hospital 011

Participants contributed important insights with respect to the context in which the DigiComp Kids system would be implemented and made suggestions on how the system could be further optimized to enable efficiency and eliminate redundancies.

#### Making the System Work in Daily Life

The fourth theme generated from the participant interviews was “Making the system work in daily life.” Facets of this theme include envisioning how the DigiComp Kids system would fit into daily workflows for families and clinicians as well as the practicality of using the system in real life. For example, parents spoke of the advantage of using the DigiComp Kids system to provide a thorough report and history of the child to different care providers:

And the more I kind of say it, the more excited I get about the idea of having it on my phone. Because even just pulling up, you know going to an appointment or having to go to the hospital or even just in a complex care appointment, being like, this is what he’s currently doing. This is our history, our results history. Or I’ve taken subsequent pictures, check out this camera roll of all of the things I’ve documented for you.

Parent 003

This parent envisioned using the system as a “one-stop” documentation hub that would be accessible from their phone to share with different health care providers. Similarly, hospital-based clinicians spoke of envisioned positive changes in their workflows while using the system.

I think the live feature of chats, of getting notified when things are happening in the home is fantastic in comparison to our regular phone call where a message is left or an email where a message is left. And you only get to it at the end of the day. Whether clinicians get notified during these events that are occurring. I think it’s great.

Hospital 012

Streamlining workflows to enable families and clinicians to easily access information and engage in real time with each other was viewed as a potential advantage of the DigiComp Kids system. These real-time connections were discussed as having the potential to facilitate proactive patient care and earlier intervention in case of patient deterioration. Finally, participants again assisted in contextualizing the proposed workflows for the intervention and identifying the necessary backups and safeguards in case of unforeseen issues:

The other thing would be a power failure, if you like potentially were working in an environment like, you’d have to have a back-up. Like if it did work for your routine documentation, you’d have to have a protocol for all your back-up um, documentation. But I guess just if you don’t have working power or working internet or data, I guess through the iPad then it may not function properly.

Home care 008

#### Reflecting on Current and Future Technology Needs

The fifth and final theme generated from the interview data was participant reflections on how DigiComp Kids might fit with their current and future technology needs. One home care nurse highlighted how the DigiComp Kids system might enable families and clinicians to have a clear understanding of disease progression to enable proactive care planning:

I think the result dashboard probably would be helpful so that we can see changes over time. I’m thinking like specifically if they have some sort of progressive disease or something that affects let’s say their breathing and their respiratory system. I think it would be helpful to those numbers over time and allow people to make decisions based on those numbers.

Home care 010

The DigiComp Kids system tracks and trends patient disease progress over time, enabling families and care providers to accurately understand current needs and forecast anticipated future needs. Importantly, the system can do this without adding additional charting for families who are already busy taking care of medically complex children. One parent commented:

I do a really bad job of tracking things. And so, having something that kind of does it for me and keeps it all together in one spot. We’re one of the few families that choose not to have nursing, so I don’t even have nursing charts. So, if somebody were to ask me... I can tell you what his normal oxygen levels are, and his normal heart rate is because I see it every night. But I have no idea what his blood pressure is or anything, not a clue. Because we don’t have nursing and I just don’t keep track.

Parent 005

Ongoing tracking of disease progression was deemed an important part of providing anticipatory and proactive care, ultimately benefiting medically complex children, their families, and health care providers alike.

## Discussion

### Principal Findings

This study was conducted to assess the usability of the DigiComp Kids intervention using the Cloud DX Connected Health System. By conducting usability testing, areas of strength and opportunity can be identified in virtual health innovations before large-scale clinical implementation.

The DigiComp Kids intervention using the Connected Health System attained high usability scores across groups, with SUM scores achieved by hospital-based clinicians, family participants, and home-based clinicians placing them in the 97th, 93rd, and 80th percentiles, respectively, in relation to SUM scores across all technology industries [30]. In addition, consistent with the definition of usability by Sauro and Kinlund [22], we noted a direct positive correlation between task error-free rates and task satisfaction.

Qualitative participant feedback highlighted favorable aspects of the DigiComp Kids system, such as its ability to connect home-based and hospital-based clinicians across geographic boundaries, to eliminate inefficiencies in care processes, and to encourage more proactive tracking of disease progress and planning for future needs. In addition, the participants assisted in contextualizing the intervention with regard to their daily lives and workflows, highlighting areas where the system could be further refined to improve the fit between user needs and system requirements.

### Interpretation

The DigiComp Kids intervention outranked most reported intervention SUM scores across the technology industries. We hypothesized that part of our high usability scores stems from the DigiComp Kids intervention, which has been co-designed alongside hospital-based clinicians, family members of CMC, and home-based clinicians. By intentionally building the system with the needs of our end users in mind, the co-design process may have contributed to the intervention achieving high SUM scores across the end user groups. Of particular interest is one component of the total SUM score, the task completion score, which was 100% across all usability participants. This score is well above the average task completion rate for technologies from usability literature, which is 78% [31]. It is possible that our task completion rates were falsely inflated due to user belief bias, whereby participants believed that the task they were being asked to complete in the simulated testing environment was indeed achievable and therefore tried harder to complete the assigned task than they might have in a real-life scenario [32]. Although we cannot determine whether user belief bias influenced our task completion scores, they should be interpreted with caution.

Qualitative interview data yielded insights beyond reflections on immediate usability testing procedures. As participants were questioned about the value of using the DigiComp Kids intervention in their daily lives, many responded by transcending their immediate circumstances, reflecting on what meaning the intervention would hold for them in the short term and long term. For example, some family participants were able to envision DigiComp Kids as part of their future daily lives, with their children in worse health than they were presently. The meaning of the system for these parents seemed to lie in its ability to create a digital bridge between hospital and home for families in need of support, either now or in the future. Evaluating the utility of a system by envisioning the role of technology in one’s future circumstances is a view supported by the literature on digital technology adoption, in that users may change their perceptions of technology value and meaning over time as their circumstances change [33]. These findings highlight the need for those responsible for implementing technological innovations such as the DigiComp Kids system to interpret user feedback in context, paying particular attention to the meaning that participants place on technology in their present and future lives. Consideration of these user perspectives allows for insight into the factors that affect technology adoption, abandonment, or sustainment [34].

### Comparisons With Prior Work

The results from DigiComp Kids usability testing using the Connected Health System built on previous usability studies by highlighting the critical roles of multimethod data collection in usability testing, consideration of the critical role of human factors, and the role of virtual health systems in connecting patients, families, and clinicians across traditional geographic barriers, as detailed below.

DigiComp Kids usability testing resulted in relatively high SUM scores across the end user groups. Despite this, the interview findings gathered from end users in our study assisted in identifying areas for improvement in the DigiComp Kids intervention. As emphasized by the DigiComp Kids study participants, the process of implementing virtual health interventions requires attention to be paid to the subjective needs of end users, together with the goals of the intervention and system requirements. These subjective needs are often best gathered using qualitative techniques, which help distill user experience data essential to assessing end user acceptance. This finding aligns with the literature, in which subjective user needs gathered via qualitative techniques illuminate distinct and important insights into end user acceptance. For example, in a study on the comparative effectiveness of 3 virtual health media for communicating health information to parents, the authors found no significant differences in the knowledge retention or efficiency of parents using each of the 3 tools; however, subjective feedback revealed a strong preference for one tool over the other 2. Similar to the qualitative results generated by family members in DigiComp Kids, parents in this study desired a tool that was simple, trustworthy, efficient, and provided practical information on condition management [35]. These qualities were important to parents in this study and were found to influence parents’ perceptions of the usability of different media, separate from quantitative metrics of usability that were collected [35]. This study highlights the importance of gathering both quantitative and qualitative information from end users, as each may offer different insights into end user preferences and overall usability. In our study, gathering qualitative experience data helped contextualize the intervention and raised issues for system improvement that otherwise may not have been uncovered until full-scale implementation.

A second important finding of the DigiComp Kids usability testing was the critical role of human factors in virtual health system usability. Human factors, or the ways in which people interact with technology, have a critical impact on the success of virtual innovations [36]. In our study, participants engaged in 2 tasks wherein icons or functions that they were required to access were hidden, either within another icon on the tablet or in a different section of the clinician’s portal. For instance, in task 7 for family and home-based clinicians (find and up-to-date medication lists), the folder that participants were required to locate was hidden within another folder. Conversely, all other icons that the participants were asked to find were accessible through paths from the main screen. Similarly, when hospital-based clinicians undertook task 8 (changing the schedule of the Wellness Surveys to be sent to participants), they needed to navigate from the individual patient profile to the main hospital clinician portal before being able to access the survey scheduling function, whereas all other clinician tasks were accessible through the individual patient profile. These 2 tasks resulted in high error rates, and low satisfaction ratings were corroborated by frustration voiced by participants in the qualitative interviews. Similar results have been reported in other usability studies. For example, in a study in which the authors tested the usability of patient portals for parents of children with chronic diseases (ie, cystic fibrosis, diabetes, and arthritis) using scenario-based usability testing and think-aloud protocols, high error rates and low completion scores resulted when information was located in a different place than participants expected [37]. Similarly, a systematic review examining the usability of eHealth interventions for adolescents with juvenile idiopathic arthritis found that adolescents using an iPod touch to input pain data made more errors, required more time per task, and reported that the device had a lower satisfaction rating than either computer-assisted or paper-based data entry [38]. This direct relationship between system ease of use and task performance emphasizes the importance of understanding human factors within formative usability testing procedures when innovating in the virtual health sphere [34].

Finally, of particular importance to DigiComp Kids participants was their ability to connect with remote clinicians in different geographic locations. In a similar usability study, McGillion et al [39] examined the usability of a postoperative hospital-to-home remote automated monitoring intervention and found that being able to connect remotely with care team members was invaluable, particularly for patients experiencing acute recovery. As was the case in our study, patient participants expressed a sense of security in knowing that a clinician would be able to monitor their postoperative progress and be reachable, should it be required [39]. Similarly, in a study evaluating an online symptom monitoring intervention for families of children with life-limiting illnesses, the authors reported increased parental empowerment over time in the study, demonstrating the value of digital technologies in supporting parents caring for their children at home [40]. This theme reinforces the important role that virtual health technologies can play in transcending traditional barriers to providing and receiving health care, such as physical location, while offering patients and families additional support in their home environments. This point may be particularly important for patients and families with frequent or intensive health care requirements, such as those with complex chronic conditions.

### Limitations

One potential limitation of this study was the lack of diversity in the participant samples. All participants in this study were English-speaking females, and the majority were White. Although the prevalent groups included in this study (ie, primary caregivers for children and nurses) have historically been predominantly female, the inclusion of male participants may have yielded different results. The inclusion of non–English-speaking participants was not possible at the time of this study because of the limitations of the study team. In addition, family participants were well educated and at least “somewhat experienced” using tablet technology; thus, our results may not be reflective of family members with lower education or less experience with tablet computing. Although the objective of a usability testing study is not to generalize results to a broad population but to uncover areas of usability strength and opportunities to make refinements, it is possible that the inclusion of a more diverse group of participants would have yielded different results and perspectives.

Another potential limitation of this study is that the collection of data related to the SUM metric and its components may have missed information that other scales capture. Our choice to collect data on effectiveness, efficiency, and satisfaction was guided by the accepted usability standards [14], which are well captured in the SUM. However, other scales, such as the Net Promoter score [41] and the Standardized User Experience Percentile Rank Questionnaire [42], capture data that we did not, such as participant ratings of trust and credibility of the intervention. We are confident that some of these data were captured in qualitative interviews; however, we did not collect quantitative data related to them.

Finally, the task completion rates across all groups were 100%, as presented in Tables 4-6. This may represent a user belief bias in our results, such that participants may be more likely to believe that the task they are being asked to complete is indeed achievable in a simulated testing scenario. Alternatively, because participants were trained in the DigiComp Kids system use immediately before usability testing, perfect task completion scores may simply represent increased training material retention by participants because of the short interval between training and testing times.

### Conclusions

The implementation of virtual health system solutions for CMCs and their families is an important initiative in providing comprehensive care in the home setting. This usability testing study offered valuable insights into the preclinical implementation phase of the DigiComp Kids intervention using the Connected Health System. Examples of such insights include the importance of tailoring the implementation of the system to match individual user needs, streamlining system features in key areas to allow for intuitive system use with the fewest steps required to complete tasks, and investigating and considering the meaning attached to system use by participants to allow for insight into system adoption and sustainment. Taken together, these findings emphasize the importance of formative virtual health system testing to uncover challenges early and refine interventions to suit the needs of end users.

## Appendix

Multimedia Appendix 1 Hospital usability testing tasks.

Multimedia Appendix 2 Usability testing patient case.

Multimedia Appendix 3 Usability tasks for home-based clinicians and family members.

Multimedia Appendix 4 Interview guide.

Multimedia Appendix 5 Formulas for usability metrics.

## Abbreviations

CMC: children with medical complexity
SUM: Single Usability Metric
