# Supplementary material for: A Novel Hospital-to-Home System for Children With Medical Complexities: Usability Testing Study
Source: JMIR Form Res. 2022 Aug 12;6(8):e34572. doi: 10.2196/34572 (PMC9419046; doi:10.2196/34572)
Supplement: Multimedia Appendix 1 [file formative_v6i8e34572_app1.docx]

**Appendix 1: User testing tasks for hospital-based clinicians**

**Task 1**

**Script:** First, I would like you to read me Emma’s last recorded heart rate, temperature, and oxygen saturation, and let me know if any alerts were generated as a result of those vital signs.

**Task:** Verbalize vital signs readings and any generated alerts for last assessment

**Steps:**

- Navigate to Emma’s profile on the Dashboard
- Correctly read out last set of vital signs assessments, including heart rate, temperature, oxygen saturation
- Correctly verbalize the alerts generated for Emma’s heart rate and oxygen saturation

**Task 2**

**Script**: Next, we are going to change the incorrect heart rate parameters for Emma, so as to avoid ‘false positive’ flags in the future. I would like you to change the ‘low’ heart rate from 60 to 80 bpm, and the ‘high’ heart rate from 100 to 130 bpm. I can repeat the values for you, if needed.

**Task**: Change Emma’s heart rate parameters from [0 (low) to 80 (high)] bpm, to [90 (low) to 130 (high)] bpm

**Steps**:

- Select ‘Notifications’ tab
- Select either bradycardia or tachycardia heart rate parameter
- Change low or high heart rate parameters to correct values
- Repeat for opposite parameter
- Click ‘Update’

**Task 3**

**Script:** Next, you receive a phone call from Emma’s mom to notify you of the low oxygen saturation reading and request a video call to assess. Please start a video call with Emma’s mother, and respond to the low oxygen saturation reading as you would normally. I will read you the credentials you will need to start the video software when needed.

**Task:** Request a video call with Emma’s family

**Steps:**

- Navigate to patient header
- Select the ‘Video’ tab
- Select ‘Start’ to submit call request
- Login to Zoom
- Connect with family and offer clinical advice

**Task 4**

**Script**: Now that you have completed a video call with Emma’s family, I would like you to add a note to the oxygen saturation reading that generated an alert from this morning.

**Task**: Add a note to the oxygen saturation reading from this morning

**Steps**:

- Navigate to Dashboard
- Click on the Bell symbol beside the most recent set of vital signs
- Type note in blank field about clinical actions taken to address the alert
- Click ‘Add note’

**Task 5**

**Script:** Finally, to wrap up this task, I would like you to document a focused respiratory assessment in Emma’s chart using the ‘Documents’ function.

**Task:** Add assessment to chart with actions taken

**Steps:**

- On screen, open Word document with assessment template
- Document a focused respiratory assessment in the appropriate field
- Save Word document to computer
- Navigate to ‘Documents’ section
- Click ‘Add Clinical Document’
- Name file and choose file to upload from computer
- Select ‘Upload’

**Task 6**

**Script**: You want to keep an eye on how Emma is doing today and decide to give her mom a call this afternoon. I would like you to schedule a video call with Emma’s family in 4 hours.

**Task**: Schedule a video call with Emma’s family in 4 hours

**Steps**:

- Navigate to Emma’s Patient Header
- Click the ‘Schedule’ button tab
- Select appropriate time slot in 4 hours
- Select ‘Video call’ under ‘Type’
- Set recurrence to ‘Once’
- Click ‘Save’

**Task 7**

**Script:** Next, you want to ensure that the Complex Care Team knows that Emma is experiencing increased acute care needs. I would like you to change Emma’s clinician-assigned risk stratification to ‘Medium’ in her Care Plan under ‘Documents’.

**Task:** Change Emma’s risk stratification to ‘Medium’

**Steps:**

- Navigate to the ‘Documents’ section
- Download the most recent ‘Care Plan’ document
- Change the ‘Care Plan’ risk stratification to ‘Medium’
- Write a note about Emma’s decreased SpO2 and increased supplemental oxygen needs under the ‘Explanation’ field
- Save the ‘Care Plan’ document with the appropriate date in the file name
- Upload the new ‘Care Plan’ document to ‘Documents’

**Task 8**

**Script**: Next, I would like you to change the frequency of Emma’s Wellness Survey from ‘Every week’ to ‘Every day’ for a period of 5 days, starting tomorrow.

**Task**: Change the ‘Wellness Survey’ from being sent once weekly to being sent every day for five days

**Steps**:

- Navigate to the Dashboard
- Select the ‘Surveys’ tab
- Select the ‘DigiComp Kids Wellness Survey’
- Select tomorrow’s date for the start time
- Set the survey to recur ‘Daily’ and end after ‘5 Occurrences’
- Select ‘Emma’ from the patient list
- Select ‘Save Schedule’

**Task 9**

**Script**: Finally, you want to let Emma’s family know that Wellness surveys will be delivered daily for 5 days in order to keep a close eye on her status. Please send a chat message to notify them of this, and a reminder to notify the Clinician on Call for any status changes.

**Task**: Send a chat message to Emma’s family

**Steps**:

- Navigate to Emma’s Dashboard
- Click on the ‘Chat’ tab
- Write a message letting Emma’s family know that Wellness Surveys will be delivered daily for 5 days, and to contact the Clinician on Call for any status changes
- Select ‘Send’
