# Supplementary material for: A Novel Hospital-to-Home System for Children With Medical Complexities: Usability Testing Study
Source: JMIR Form Res. 2022 Aug 12;6(8):e34572. doi: 10.2196/34572 (PMC9419046; doi:10.2196/34572)
Supplement: Multimedia Appendix 2 [file formative_v6i8e34572_app2.docx]

**Appendix 2: Usability Testing Patient Case**

***Introduction to case***

As you probably noticed on your Clinician Dashboard, you have a patient assigned to you for today – Emma. Emma is a 2.5-year-old girl with Spinal Muscular Atrophy Type I (SMA1). As a review, SMA is a genetic disorder that results in progressive muscle weakness and paralysis. Children with SMA1 do not typically live past 4 years of age.

Emma was diagnosed at 8 months old at the Children’s Hospital when her parents became concerned that she was failing to meet developmental milestones such as holding her head up and grasping objects.

Let’s review some of Emma’s most important health issues. Emma has a home oxygen saturation monitor that shows acceptable oxygen levels when she is awake, however, she has started to require low-flow oxygen of 0.5-2 L for nights and naps. She also requires intermittent suctioning on a regular basis, and more often when she is sick. Emma’s parents are aware that she will likely soon require a form of positive pressure ventilation for sleeping and possibly continuously as the disease progresses. Emma’s parents are very engaged with her care but exhausted and still trying to learn how to manage all of her symptoms. Emma’s family has been given a DigiComp Kids Kit to help them manage at home.

***Progression of case*** (between tasks 2 and 3)

*Moderator will connect with clinician via phone call, acting as Emma’s mom. Moderator will relay that Emma seems to be a bit ‘stuffy’ lately, and it has been difficult to keep the nasal prongs in her nose overnight with the increased congestion.*

***Progression of case*** (between tasks 5 and 6)

So, to continue our patient scenario, I will give you a quick update. After your assessment of Emma, you spoke with the rest of the Complex Care Team, and decided to continue to monitor Emma at home, checking in at least once a day with family using a video call, or more frequently if needed.

Emma’s mom re-checked Emma’s vital signs toward the end of her nap this morning, and they read as follows:

HR: 118 bpm (asleep)

SpO2: 95% on 3L O2 (nasal prongs)

Resp: 22 (asleep)

T: 37.8 (axilla)

Emma was relaxed and sleeping peacefully, with no respiratory distress noted.
