# Supplementary material for: A Novel Hospital-to-Home System for Children With Medical Complexities: Usability Testing Study
Source: JMIR Form Res. 2022 Aug 12;6(8):e34572. doi: 10.2196/34572 (PMC9419046; doi:10.2196/34572)
Supplement: Multimedia Appendix 3 [file formative_v6i8e34572_app3.docx]

**Appendix 3: User testing tasks for home-based clinicians and family members**

**Task 1**

**Script:** Ok, so the first step that I would like you to do is set up the tablet, by plugging it in and turning it on.

**Task:** Set up and turn on the tablet

**Steps:**

- Open the tablet stand and place tablet on stand
- Place the lava blue adapter box to the Velcro® on stand
- Connect the small black connecting cord to the tablet in the micro-USB/charging port and lava blue box.
- Attach the power cord to the lava blue adapter box and electrical outlet.
- Turn on the tablet by pressing and holding the small rectangular button on the side of the tablet.

**Task 2**

**Script:** Next, please unlock to the tablet by swiping a large letter “C” across the screen. Then, I will read you your username and password, which I would like you to enter into the tablet in order to login. [When participant has unlocked the tablet, read them their user ID: XXX-XXX, and password: XXX-XXX-XXXX].

**Task:** Login to the tablet

**Steps:**

- Create a letter “C” on the screen by swiping right to left across the top, down the left side, and left to right across the bottom
- Enter the specified 6-digit username on the screen
- Enter the specified 10-digit password on the screen

**Task 3**

**Script:** Next, I would like you to determine if there are any available measurements or surveys to take, and how you know that they are available or unavailable.

**Task:** View and interpret pending measurements and surveys using red asterisk

**Steps:**

- Correctly identify that three measurements (oxygen saturation, heart rate, and temperature) are available, as indicated by the red asterisk
- Correctly identify that one survey (‘Wellness Survey’) is available, as indicated by the red asterisk

**Task 4**

**Script:** Now that you have determined that a temperature measurement is due soon, I would like you to take/describe the steps to take [name of child]’s temperature using the appropriate device.

**Task:** Complete/describe temperature measurement

**Steps:**

- On tablet, select “take a reading”, then “body temperature”
- Turn on the thermometer
- [Ear] Place the end of the thermometer in ear canal. Press the button on the top of the thermometer to take the reading.
- When you have heard the thermometer “beep”, the reading will be visible on the digital screen on the thermometer.
- Select “manual entry” on tablet and enter the measurement

**Task 5**

**Script:** Please also apply/describe the application of the appropriate device to [name of child] and complete an oxygen saturation measurement.

**Task:** Complete/describe oxygen saturation measurement

**Steps:**

- On tablet, select “take a reading”, then select “pulse oximetry”
- Turn on the oximeter
- Place the oximeter on one of the child’s finger
- The tablet will prompt when the reading is sufficient and ready to send the reading to the database

**Task 6**

**Script:** Next, I would like you to complete the Wellness Survey that is due, and send it to the hospital-based clinical team for review.

**Task:** Complete and submit Wellness Survey

**Steps:**

- Select “Surveys” from the main menu
- Select “Wellness Survey”
- Answer the questions by selecting the appropriate bubbles on the screen
- Press “Send the survey” once you have completed it

**Task 7**

**Script:** Next, I’m going to ask you to read me the most up-to-date list of the child’s medications.

**Task:** Find and read out the up to date list of child’s medications

**Steps:**

- From the main menu of the tablet, click on “Documents”
- Select the most recent document, titled “Medication Record”
- Correctly read out the list of the child’s most recent medications

**Task 8**

**Script:** Finally, I would like you to use the tablet to send a photo to the hospital-based clinical team. For our purposes today, you may take a photo of any inanimate object in your environment, such as your keyboard, desk, or a pen.

**Task:** Take and submit a photo using the tablet

**Steps:**

- From the ‘Home Screen’ select ‘Take a Reading’
- Select the ‘Photo’ icon
- Once the camera loads, focus the camera on the object and tap the white circle on the screen.
- The photo will automatically be loaded to the patient’s profile.
