# Supplementary material for: A Novel Hospital-to-Home System for Children With Medical Complexities: Usability Testing Study
Source: JMIR Form Res. 2022 Aug 12;6(8):e34572. doi: 10.2196/34572 (PMC9419046; doi:10.2196/34572)
Supplement: Multimedia Appendix 4 [file formative_v6i8e34572_app4.docx]

**Qualitative Debrief Interview Guide**

**Opening**

1. Thank you once again for participating in our user testing sessions! To begin, please tell me about your experience using the *DigiComp Kids* system.
   - *Prompts: How did you find the overall process? Was there anything unexpected about this experience for you?*

**Theme: Technology**

I am now going to ask you about the technology features of the DigiComp Kids system. By technology features, I mean the digital devices and/or software that you were asked to use during your training and testing sessions.

1. If you had access to the technology features that you used during your training and testing sessions in real life, do you think it would change the way that you provide care to medically complex kids? Please tell me about that.
   - *Prompts: What specific technology features do you think are most important?*
2. If anything, which technology features need to be changed in *DigiComp Kids* to make it better for healthcare providers and families of medically complex kids to use?
   - *Prompts: Did you struggle with any specific device or task? Is there something that can be added or changed to make the DigiComp Kids system easier to use?*

**Theme: People**

I am now going to ask you to think about using the *DigiComp Kids* system to connect with other important people who help to provide care for [your medically complex child/medically complex kids], in your daily life – we will call these people your “team members”.

1. Would you use the *DigiComp Kids* system to connect with other team members? How would you do this? What are the possible benefits and disadvantages of using the *DigiComp Kids* system to connect with other team members?
   - *Prompts: Who would you connect with most? Are there specific team members that you can think of?*

**Theme: Context**

This last section focuses on how the *DigiComp Kids* system might fit with your daily life and routines.

1. During your user testing session, was there anything in your environment that made it easier or more difficult to use *DigiComp Kids*? Please tell me about that.
   - *Prompts: Consider the physical environment, as well as your social, work, and/or family environments.*
2. How would *DigiComp Kids* fit into your daily life and routines caring for [your medically complex child/medically complex kids]? Are there ways that we can change the *DigiComp Kids* system to make it fit better with your daily life?
   - *Prompts: Would you need to alter your routines to use DigiComp Kids?*

**Closing**

1. Is there anything else that you would like to tell me about your experience with the *DigiComp Kids* system?
