# Supplementary material for: A Novel Hospital-to-Home System for Children With Medical Complexities: Usability Testing Study
Source: JMIR Form Res. 2022 Aug 12;6(8):e34572. doi: 10.2196/34572 (PMC9419046; doi:10.2196/34572)
Supplement: Multimedia Appendix 5 [file formative_v6i8e34572_app5.docx]

**Appendix 5: Formulae for Usability Metrics**

### Task Completion

Task completion is determined by coding completed tasks as 0 (task incomplete) or 1 (task completed). The task completion calculation is then performed by computing the ratio of successful task completions to task attempts. The following calculation describes the measurement of task completion:

$\frac{Successful task completions}{Task attempts}$ *= task completion rate*

### Error rates

Task-level error rates are also measured using discrete data and computed as a ratio. However, an additional step is needed in order to define the opportunities for errors to occur with each task. By defining the opportunities for errors, or “error potential”, a reliable denominator for each task is established [1]. More complex tasks are likely to have more error opportunities, while simpler ones are less susceptible to user error commission. Therefore, one cannot simply use “task attempts” to define “opportunities”. Instead, total error opportunities for a given task are defined as the number of sub-tasks that a user must successfully complete in order to accomplish a given task [2]. Multiple errors are possible with each sub-task, therefore, errors are “counted” for each failed sub-task attempt, regardless of how the error was committed.

To calculate the error rate for a task, the total number of errors committed are divided by the total error opportunities (the number of sub-tasks multiplied by the number of participants attempting each sub-task). The error-rate is then subtracted from 1 to get the error-free rate.

The following formula is used to define the task-level error rate:

$\frac{Total task errors committed}{(Number of participants x number of sub-tasks)}$ = *error rate*

### Task times

To compute task times as a factor of usability, an ideal task time must first be established. While establishing an ideal task time requires a certain level of arbitrariness, one logical starting point is comparing the task time of user testing participants to that of an expert system user. In the DigiComp Kids study, the expert user was the lead author (MB). To set a specification limit for acceptable task times, Sauro and Kinlund suggest multiplying the time of the expert user by 1.5 [3].

Next, the task times of user testing participants (measured in seconds) are standardized by converting them to a Z score using the normal distribution curve, so that they may be combined with other usability metrics in a single, summative score. The calculation for the Z score is given below:

$\frac{Sample mean-specifiction limit}{Sample standard deviation}$ = *task time Z score*

Since lower task times are desirable, the Z score is then used to find the corresponding area on the normal distribution curve, and this area is then subtracted from one.

### Satisfaction scores

Finally, similar to the continuous nature of task time data, satisfaction scores may also be treated by calculating a Z score. To measure task satisfaction in the DigiComp Kids study, we used the Single Ease Question (SEQ)— a seven-point system for rating how easy or difficult users find a task. Using the SEQ, the limit for unacceptable satisfaction scores has been shown to be scores below 5.6 on the 7-point scale [2, 4]. The calculation for the Z score is shown below:

$\frac{Sample mean-specifiction limit}{Sample standard deviation}$ = *satisfaction Z score*

Since higher satisfaction scores are desirable, the area under the normal curve that corresponds with the calculated Z score is found and used as a standardized satisfaction level.

1. Sauro, J. and E. Kinlund. *A Method to Standardize Usability Metrics Into a Single Score*. in *SIGCHI Conference on Human Factors in Computing Systems*. 2005. Portland, Ore.: ACM.

2. Sauro, J. and E. Kindlund, *Making Sense of Usability Metrics: Usability and Six Sigma.* Computer Science, 2005.

3. Sauro, J. and E. Kinlund, *How Long Should a Task Take? Identifying Specification Limits for Task Times in Usability Tests*, in *HCI 2005*. 2003: Las Vegas, Nevada, USA.

4. Nielsen, J. and J. Levy, *Measuring Usability: Preference vs. Performance.* Communications of the ACM, 1994. **37**: p. 66-76.
